# A Novel Three-Phase Model of Brain Tissue Microstructure

**DOI:** 10.1371/journal.pcbi.1000152

**Published:** 2008-08-15

**Authors:** Jana L. Gevertz, Salvatore Torquato

**Affiliations:** 1 Program in Applied and Computational Mathematics, Princeton University, Princeton, New Jersey, United States of America; 2 Department of Chemistry, Princeton University, Princeton, New Jersey, United States of America; 3 Princeton Institute for the Science and Technology of Materials, Princeton University, Princeton, New Jersey, United States of America; 4 Princeton Center for Theoretical Physics, Princeton University, Princeton, New Jersey, United States of America; Indiana University, United States of America

## Abstract

We propose a novel biologically constrained three-phase model of the brain microstructure. Designing a realistic model is tantamount to a packing problem, and for this reason, a number of techniques from the theory of random heterogeneous materials can be brought to bear on this problem. Our analysis strongly suggests that previously developed two-phase models in which cells are packed in the extracellular space are insufficient representations of the brain microstructure. These models either do not preserve realistic geometric and topological features of brain tissue or preserve these properties while overestimating the brain's effective diffusivity, an average measure of the underlying microstructure. In light of the highly connected nature of three-dimensional space, which limits the minimum diffusivity of biologically constrained two-phase models, we explore the previously proposed hypothesis that the extracellular matrix is an important factor that contributes to the diffusivity of brain tissue. Using accurate first-passage-time techniques, we support this hypothesis by showing that the incorporation of the extracellular matrix as the third phase of a biologically constrained model gives the reduction in the diffusion coefficient necessary for the three-phase model to be a valid representation of the brain microstructure.

## Author Summary

The goal of the present work is to develop a biologically constrained three-dimensional model of the brain microstructure. This is an important task because the brain's three-dimensional microstructure cannot be directly visualized, yet a knowledge of its structure is essential for understanding normal brain functioning. We first explore the shortcomings of the conventional modeling approach that treats brain tissue as a two-phase material. These models either do not preserve realistic features of brain tissue or preserve these properties while overestimating the brain's effective diffusivity, an average measure of the underlying microstructure. We thus developed a biologically constrained two-phase model that, upon analysis, achieves a lower diffusion coefficient than other constrained models yet proves to not have a low enough diffusion coefficient to be a valid representation of the brain microstructure. We then show that if the extracellular matrix is incorporated as a third phase in this model, then the reduction in the diffusion coefficient achieved allows the proposed model to be a valid representation of the brain microstructure. Using this model, we can test the impact that microstructural changes have on the transport of nutrients and signaling molecules in the brain.

## Introduction

Brain tissue is naturally divided into two domains, the intracellular space (ICS) and the extracellular space (ECS). The ICS is a tightly packed composite of neurons, glia and their cellular extensions, and the ECS is the microenvironment that separates brain cells [1]. The structural maintenance of the ECS is essential for normal brain functioning, as intercellular communication, nutrient transport and drug delivery all depend on ECS integrity. Many pathological brain conditions are associated with changes in ECS size and geometry, including ischemia, inflammation, and tumor progression [2].

Given that the brain is naturally divided into these distinct regions, its microstructure can be well-described as a *random heterogeneous material*, a medium that is composed of randomly arranged domains of different phases [3]. Brain tissue has conventionally been modeled as a two-phase material, where the two domains are the ICS and the ECS. Despite the seemingly natural classification of the brain as a two-phase material, it is important to note that the ECS is actually a heterogeneous composite of ions, transmitters, metabolites, peptides, neurohormones and molecules of the extracellular matrix (ECM) [2]. Using current imaging techniques, the ECS can be visualized in two dimensions, but not in three dimensions. An electronmicrograph done by Dr C.B. Jaeger of a small region of rat cortex can be found in [4].

According to the theory of random heterogeneous materials, macroscopic properties of a medium provide an average measure of the underlying microstructure [3]. This is particularly useful in the case of brain tissue, since detailed three-dimensional (3D) microstructural images do not exist, but macroscopic properties can easily be measured. The ECS volume fraction *ϕ*_1_ and effective diffusion coefficient *D*_e_ are the two macroscopic parameters commonly employed to give an average description of brain's microstructure [2]. In fact, diffusion analysis can be applied to study the the microstructure of any tissue type [5]. For brain tissue in particular, the real-time iontophoretic (RTI) method has been applied to determine macroscopic properties. Using tetramethylammonium (TMA^+^) as the tracer, it has been measured that *ϕ*_1_ = 0.2 and *D*_e_ = 4.8×10^−6^ cm^2^ s^−1^ [1]. More commonly, one represents the effective diffusion coefficient using one of two dimensionless quantities: the dimensionless effective diffusivity, defined as D* = *D*_e_/*D*_1_, or tortuosity, defined as *λ* = (*D*_1_/*D*_e_)^1/2^, where *D*_1_ is taken to be the diffusion coefficient of TMA^+^ in agarose [1],[2]. We note here that in the field of random heterogeneous materials, tortuosity is defined as *λ* = *D*_1_/*D*_e_ [3]. The definition used here is consistent with that used by others studying diffusion in brain tissue. It has been measured that *D*_1_ = 1.2×10^−5^ cm^2^ s^−1^, giving a brain tortuosity of *λ*≈1.6 and a dimensionless effective diffusivity of D*≈0.40. In this paper, all results will be presented in terms of D*. Volume fraction and diffusion measurements that differ significantly from these average values are hallmarks of pathological brain states, highlighting that these macroscopic parameters capture significant microstructural information. Nonetheless, these parameters cannot fully describe the underlying microstructure, as this can only be done via an infinite set of *n*-point correlation functions [3].

Armed with information about the brain's macroscopic properties, theoretical models of the microstructure can be developed. Previous attempts have been made to model the brain microstructure as a two-phase isotropic material composed of uniformly spaced closely packed convex cells [6]–[10]. In the studies that simply treat brain cells as homogeneous impenetrable obstacles, it has been predicted that the effective diffusivity of brain tissue is well-approximated by the two-phase Hashin–Shtrikman (HS) upper bound
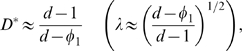
(1)where *d* is the spatial dimension [3], [6]–[9]. At ECS volume fraction *ϕ*_1_ = 0.2, the 3D HS bound predicts that D*≈0.71, a diffusivity significantly larger than that measured in brain tissue. For this reason, it is clear that two-phase models composed of uniformly spaced closely packed convex cells lack some key features of brain tissue.

Recent work in ischemic brain tissue suggests that dead-end microdomains are a major determinant of extracellular tortuosity, although it is currently unknown if such voids and dead-ends exist in normal brain tissue [11]. Since dead-ends have been implicated in ischemia, several approaches have been taken to incorporate dead-ends into two-phase models of healthy brain tissue. In one approach, dead-ends arise because cubic brain cells are allowed to overlap. An unrealistically large number of concavities are found when cubic cells can overlap, giving diffusivities significantly below that measured in brain tissue [12]. In another approach [6],[13], rectangular dead-end cavities (open at one end to the ECS) are punched into convex cellular elements. While the technique can yield a model with the measured diffusivity of brain tissue, brain cell bodies are generally described as being convex objects with fine cellular extensions emanating from the body [14], not a geometry consistent with representing brain cells in this manner. While the notion of cellular convexity has not been verified for all cells, there is certainly no biological evidence suggesting that concavities are found in all brain cells. Thus, given the known features of brain cells, including the lack of evidence that an abundance of concavities exist in brain cell bodies, it is reasonable to assume that concavities play a role in, but are not the sole factor responsible for the diffusivity of brain tissue being significantly smaller than that predicted by models of uniformly spaced convex cells.

If we work under the assumption that the majority of brain cell bodies are convex, alternate mechanisms need to be implemented to develop a realistic microstructural model that preserves the topological and geometric features of the ICS and ECS while simultaneously having the correct diffusion properties. Designing an appropriate microstructural model for tissue in general is tantamount to a packing problem; i.e., dense aggregates of cells or “particles” [3], [15]–[19]. Approaching model development from this perspective lends further support to the notion that most brain cells are convex, as tightly packing these cells in space is a densification procedure, and such processes tend to drive the shape of the object being packed towards convexity. Given the low porosity and diffusivity of brain tissue, the nonoverlapping mostly convex cells of the ICS have no choice but to pack tightly. With the appropriately chosen packing procedure, realistic geometric features of brain tissue will naturally emerge. However, it is important to note that when nonoverlapping convex cells are packed in 3D space, very few dead-ends actually arise. This is because topological connectedness increases with dimension [3], and although packings of convex cells in 2D can result in a significant amount of dead-end space, this is not the case in 3D. Based on this observation, we conclude that an insufficient amount of dead-end space naturally arises because of the size, shape and distribution of brain cells. This does not rule out the possibility that other factors, such as glial cell processes or ECM macromolecules, can lead to the formation of dead-end microdomains in the brain, but it does highlight that current models are not appropriately accounting for a dominant mechanism that contributes to brain tissue tortuosity.

In this paper, we propose a novel three-phase model of the brain microstructure that obeys known properties of the ECS and ICS, naturally develops a small number of dead-end microdomains due to cell shape and position, and accounts for diffusion hindrance by ECM macromolecules. In light of the highly connected nature of 3D space, as well as the previously mentioned fact that the ECS, unlike water, is a restricted medium for diffusion [20], we hypothesize that the inclusion of the ECM as the third phase in our proposed model can give the decrease in the diffusion coefficient necessary for the model to have the same diffusivity as brain tissue. To confirm that the model is a realistic representation of the brain microstructure, diffusion properties of the medium both with the ECM (three-phase model) and without the ECM (two-phase model) are studied using an accurate state-of-the-art technique borrowed from the field of random heterogeneous materials: a first-passage-time Monte Carlo simulation [21].

We find that by adding the ECM to our two-phase model (which acts to reduce the free diffusion coefficient of the ECS in accordance with previous experimental observations [20]) the model can achieve an order of magnitude decrease in the diffusion coefficient and, at the appropriate ECM concentration, conforms to the diffusion properties measured in brain tissue. From this study, we argue that two-phase media subject to a proposed set of biological constraints, which includes limiting cells to be mostly convex bodies, cannot achieve the diffusion parameters of brain tissue. We have shown that, as suggested from experimental data, the addition of the ECM gives the decrease in the diffusion coefficient necessary for the model to conform to the macroscopic properties of brain tissue. It is plausible that the novel arrangement of cells proposed herein, along with the implementation of the ECM, can further benefit by incorporating more dead-ends. Nonetheless, the contribution of this work is to highlight the importance of, and suggest a method to implement ECS heterogeneity in realistic brain microstructural models.

## Results

### Properties of Brain Microstructure

To accomplish our goal of developing a realistic microstructural model, we have compiled a list of brain tissue features:

P1 The cells and cellular processes of the ICS are mostly convex and densely packed [1],[9],[14]. While detailed morphological studies need to be done to rigorously verify the convexity of brain cell bodies, the existing data does not suggest that a large number of brain cell bodies contain concavities.
P2 Neuron and glial cell bodies can range anywhere from 10 to 80 µm in diameter [22]. Cellular processes can have cross-sections as small as a fraction of a micrometer [14].
P3 All cells of the ICS are surrounded by ECS. The size of the ECS around each cell, as measured using integrative optical imaging of diffusing dextrans and water-soluble quantum dots, varies between 38–64 nm [23]. This estimate of ECS width is several times larger than the prediction of 10–20 nm made using electron micrographs of fixed adult brain tissue sections. However, measurements made using electron micrographs are thought to underestimate ECS width, as the ECS in analyzed sections are likely to have contracted due to ischemia [23].
P4 The ECS occupies 20% of the total brain volume [1].

Despite the semipermeable nature of cell membranes, in our model we assume that all cells of the ICS are impenetrable. The development of a realistic microstructural representation of brain tissue is independent of the permeable nature of the ICS, and therefore this simplification is justified.

Another important feature of the brain microstructure is the cellular processes that emanate from cell bodies. Axons and dendrites, the processes that arise from neuronal cell bodies, are generally convex structures with vastly different morphologies. Axons are typically a single long cylindrical structure, whereas dendrites are branching cylindrical structures [1]. Glial cell processes are thin sheet-like structures that exhibit a wide range of morphological variability. While these processes are certainly important in the brain, the complexity of their structures makes it very difficult to incorporate them into a brain microstructural model. It is certainly plausible that allowing some cellular concavities and dead-ends to persist in the model may grossly account for these features, as has previously been tackled [6],[13]. Without denying the validity of this approach, our goal here is to limit the number of concavities and dead-ends (sticking with the assumption of mostly convex cellular bodies) and yet develop a realistic microstructural model with the correct diffusion properties.

### Two-Phase Model of Brain Microstructure

In order to develop our three-phase model, we begin by proposing a novel two-phase model that accounts for the four properties of brain tissue. In lieu of property P1, the intracellular space must be composed of mostly convex objects. Previous theoretical work has concluded that convex cells of different shapes arranged comparably give rise to the same medium tortuosity [3],[9]. In particular, provided that the cells are compact convex shapes of high symmetry and have the same spatial arrangements, one can be certain the diffusion properties will be comparable even if the shapes are different [3]. If one is not careful and chooses shapes without high symmetry and then also uses a different spatial arrangement, it is then the case that the shape of the cell can influence the diffusivity of the medium. Since biological cells are higly symmetric, in order to achieve the desired porosity in our model, brain cells are represented by the most basic convex shapes: squares in 2D and cubes in 3D. We will generally use the term cube to describe both squares and cubes for succinctness. Ordered configurations of uniformly spaced cubes on a lattice have already proven to be an unsatisfactory model of the brain microstructure, as the medium is not sufficiently tortuous [9]. In an effort to develop a more tortuous model, we propose a packing construction that exhibits both brain cell size and shape variation (P2), as well as nonuniformity of spacing between brain cells (P3).

**Figure 1:**
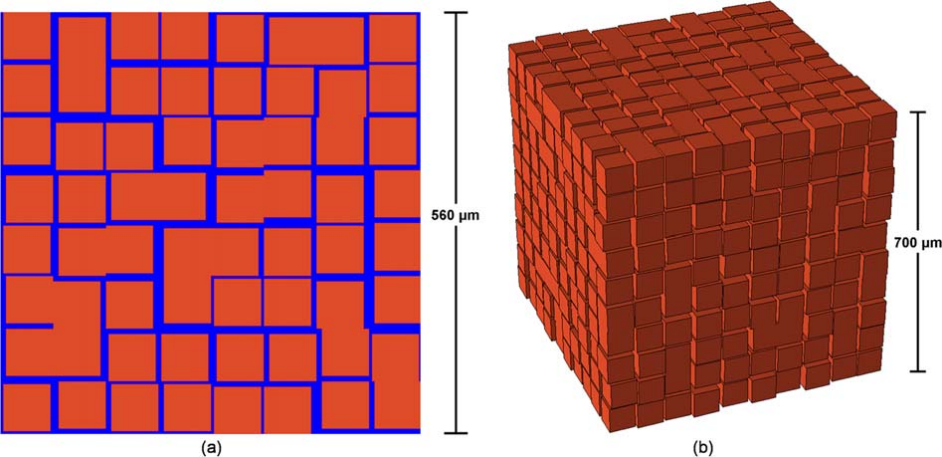
Proposed Two-Phase Model. Representative region of proposed microstructural model. (A) 2D two-phase model with ICS in red and ECS in blue. (B) 3D two-phase model (nonstaggered case) with ICS in red.

To develop our novel model of nonoverlapping and nonuniformly spaced cubes, begin by dividing space into *N*×*N* squares (2D) or *N*×*N*×*N* cubes (3D). In order to balance computational restrictions with the desire to simulate a large number of cells, *N* was taken to be 30 in 2D (giving 900 cells) and 7 in 3D (giving 343 cells). Furthermore, in 2D each square element was divided into 30×30 pixels, and in 3D each cubic element was divided into 70×70×70 voxels. These cubes can be nonstaggered, staggered in one direction, or staggered in two directions in 3D space. Within each of these regions, a “target volume” is defined. Each region is then populated with a cubic obstacle that occupies 80% of the region and has its center coordinate randomly placed in the target volume. The approach described here can be generalized to allow more variation in cell shape and size by permitting the placement of both cubical and cuboidal cellular obstacles. The resulting geometric representation of the brain microstructure (in the nonstaggered case) can be seen in Figure 1. In 3D, we are modeling approximately a 2.16×10^−3^ mm^3^ volume of brain tissue. The model will be analyzed using periodic boundary conditions to minimize boundary effects.

Properties P1–P4 of brain tissue are satisfied by the proposed two-phase model. In particular, the model is composed of densely packed mostly convex cells and the ECS occupies 20% of space. While each obstacle placed into the system has a fixed size, the placement of some obstacles results in the formation of elongated cells, some of which are oddly shaped and not convex. This feature is desirable, as not all brain cells are convex and, as property P2 states, brain cells can vary in size. Each cellular object is surrounded by ECS, and the ECS surrounding each cell is not uniform in width. Finally, because obstacles placed in each region can touch neighboring obstacles, a small number of dead-end regions naturally arise in this geometry.

### Limitations of Two-Phase Models

It is essential that we evaluate the limitations of two-phase models to justify the development of a three-phase model. Previously developed two-phase models either overestimate the brain's effective diffusivity [9], or achieve the diffusivity by introducing a large number of cellular concavities [6],[12],[13]. Using the discrete first-passage-time algorithm (see Methods section), we wanted to determine if the proposed two-phase model has a diffusion coefficient comparable to that measured in brain tissue. The model was analyzed in 2D and 3D to determine the impact dimensionality has on the results.

We found that the 2D model is a successful representation of the brain microstructure: the geometry proposed is subject to the same set of biological constraints as brain tissue and has similar diffusion properties (data not shown). While the 2D results are promising, the brain is a 3D structure. For this reason, we next applied the first-passage-time technique to test if the 3D geometry successfully reconstructs the brain microstructure (Table 1). While all 3D media created satisfy the two-phase HS bound, the lowest diffusivity obtained (D* = 0.63 in both the staggered and nonstaggered case) is significantly larger than the effective diffusivity measured in brain tissue (D* = 0.4). Even though the proposed two-phase model has a lower diffusivity than models of uniformly spaced convex cells, the 3D model, unlike its 2D analog, does not have a low enough diffusivity to be a valid representation of the brain microstructure.

**Table 1:** Diffusivity of Two-Phase 3D Models.

	D*
HS upper bound	0.71
Nonstaggered model: cubes only	0.65
Nonstaggered model: long cuboids	0.63
Nonstaggered model: short cuboids	0.63
Staggered in one direction: cubes only	0.63
Staggered in two directions: cubes only	0.63
Target	0.4

Effective diffusivities of our proposed two-phase 3D models are compared to the HS upper bound and to the experimentally observed effective diffusivity of brain tissue. Cubes have a fixed size (*L*×*L*×*L*), long cuboids are any permutation of a cuboid of size 2*L*×2*L*×*L*, and short cuboids are any permutation of a cuboid of size 2*L*×*L*×*L*.

### Three-Phase Model of Brain Microstructure

**Figure 2:**
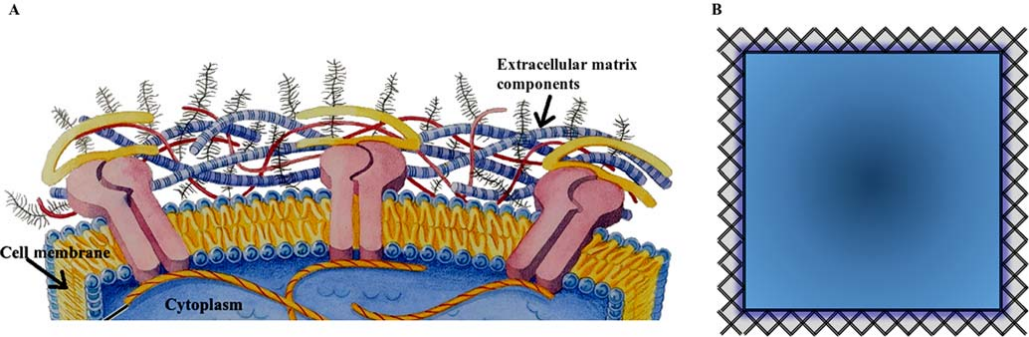
ECM Incorporation into Model. (A) Schematic representation of a cell and some of the associated ECM components. Note how the ECM forms in close proximity to the cell that produces it. Image is adapted from:http://courses.cm.utexas.edu/jrobertus/ch339k/overheads-2/figure-07-30.jpg. (B) Our representation of the ECM in the proposed three-phase model. The square represents a convex cell body and the “x-ed” network surrounding the cell represents the ECM.

In order to develop a biologically constrained model of the brain microstructure with the expected diffusion properties, we return to the experimental observation that the ECS is not a homogeneous solution, but is instead a heterogeneous composite, and the largest components in this composite are the macromolecules of the extracellular matrix [2]. By definition, the extracellular matrix is an intricate network of macromolecules that assemble into an organized meshwork in close association with the surface cell that produces them (Figure 2A) [24]. We propose that the ECM be treated as an independent third phase of the brain microstructure. While the limitations of two-phase models discussed in the previous section lead us to deviate from the conventional two-phase modeling approach, it is important to reiterate that the novelty of this work is the direct inclusion of ECS heterogeneity into a model of the brain microstructure. Although this model is thus not proposing a new biological mechanism, it is certainly guided by experimental evidence that suggests that a three-phase model fits the task at hand. Firstly it has been shown that molecular changes in ECM content occur during normal and pathological processes that are characterized by altered brain diffusion properties [2]. This observation provides evidence that there is a correlation between changes in ECM content and the diffusion of small tracers in the brain, although no causative relation has been proven. Secondly, it has been speculated that the transport of positively charged molecules (such as the tracers used in RTI experiments) is hindered by the negative charge associated with the ECM [11], lending further support to the theory that the ECM does impact the diffusion of small ions in the brain.

Given the theoretical evidence presented against conventional two-phase models and the biological evidence which suggests that the ECM may regulate brain diffusion properties, we turned the two-phase model that obeys properties P1–P4 of brain tissue into a three-phase model by introducing the ECM (the third phase) into the ECS. Introducing the ECM into the model necessitates some a priori knowledge on the concentration, diffusion properties and precise structure of the ECM in the brain ECS. The unavailability of this information [11] necessitated a minimalistic modeling approach. Given the aforementioned definition of the ECM, we can envision the ECM as a low volume fraction mesh-like network that surrounds each cell in the brain. Thus, from a modeling perspective, a logical minimalistic first assumption is that the ECM forms a “shell” around each cell (Figure 2B). This shell can act as either a barrier to diffusion, or more likely, can act to slow down diffusion near cell boundaries by trapping diffusing particles in the ECM mesh. Since it is unclear how to approach modeling this mesh-like structure and its altered diffusion properties with any accuracy, we will consider a first-order approximation to this situation. We propose that the ECM can be modeled as having the *net* effect of excluding a diffusing ion from some volume about the cell. We emphasize that volume exclusion is a net effect of the ECM, because it is more likely that the ECM reduces the diffusion coefficient in the area surrounding cells rather than excluding diffusion all together. If there were no other macromolecules in the ECS, this shell would just be a first-order approximation of the ECM mesh. However, there are other macromolecules that float around the ECS that are not formed in close association with a cell. If we want to also consider the effects of these molecules in our model, we need a computational technique that can predict the average influence of all of these molecules; that is, both those that are in close association with a cell, such as the ECM, and other freely suspended molecules.

A technique that allows us to treat the ECM as a mesh-like shell around each cell while also accounting for the effects of those ECS molecules that are not associated with a cell is to use a finite-sized diffusing particle in our first-passage-time simulations. To explain why this implicit representation of the ECM plus other ECS molecules is reasonable, consider what happens when we only consider the ECM without any other molecules in the ECS. When we allow a diffusing tracer to take on a finite size, the tracer is excluded from a larger volume fraction than dictated by cell size, and this exclusion volume is nothing more than the “shell” we defined earlier to represent the ECM. Of course, this analogy only applies if the shell fully inhibits the diffusion of small ions, which is unexpected. Thus, if we were ignoring the effects of other ECS molecules and we just focused on the ECM, this approach gives us a first-order approximation on the net effect the ECM has on diffusion. We do not claim that the shell is of the proper concentration or is modeled with the correct diffusion coefficient, just that it has the same effect as the mesh-like network with the correct volume fraction and diffusivity. Since the ECM molecules are not the only compounds found in the ECS, there is no reason this first-order approximation has to only account for the effects of the ECM. The first-order approximation we propose here actually models the net effect of both the ECM and other molecules that are found free-floating in the ECS.

**Figure 3:**
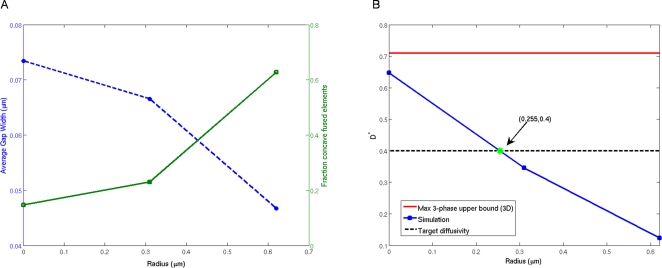
Properties of Three-Phase Model. (A) The left *y*-axis (dashed blue line with circles) gives the average gap width in the model and the right *y*-axis (solid green line with squares) gives the fraction of concave cells in the model. Both plots are given as a function of particle radius (in µm). B) Effective diffusivity of 3D three-phase media at ECS volume fraction *ϕ*_1_ = 0.2 as a function of the particle radius. The results of the simulation are compared to the maximum two-point three-phase upper bound (Equation 3 with *a* = 1; solid red line) and the target diffusivity (dotted black line).

In our first-order approximation, one key parameter, the radius of the diffusing particle used in simulations, will measure the exclusion-volume effects caused by the ECM plus other ECS molecules [25]. The larger this parameter, the more hindrance a particle encounters or the more time a particle is trapped as it diffuses through the ECS. It is important to note here that the finite-sized diffusing tracer is used to implicitly represent the presence of the ECM plus other ECS molecules; it is not related to the size of the actual tracer used in RTI experiments! In order to quantify the effects that this hard-shell approximation of the ECM has on the proposed microstructural model, we have studied how both the average gap width and the fraction of concave cells changes as a function of the diffusing particle radius, and these results are summarized in Figure 3A. We have found that, as expected, the average gap width in the model decreases as the particle radius increases. More importantly, we have quantified how the fraction of concave cells increases in our model as a function of particle radius. As seen in Figure 3A, we have found that slightly less than 15% of the cells in the two-phase model (particle radius equals zero) are concave. If the diffusing particle is allowed to have a radius of 1 voxel (which corresponds to 0.31 µm in our model), the percent of concave cells increases to 23%. Further increasing the radius to 2 voxels increases the percent of concave cells to 63%. Thus, our first-order approximation of the ECM has the net effect of decreasing the average gap width in the model and increasing the percent of concave cells.

### Significance of Three-Phase Model

The 3D first-passage-time algorithm was applied to the proposed three-phase model (all cubes; nonstaggered case), using a finite-sized diffusing particle to represent ECS heterogeneity (the presence of the ECM plus other ECS molecules). Simulations were conducted for various values of the diffusing particle radius to probe the effects of a wide concentration of ECS molecules (Figure 3B). When the radius of the diffusing particle is approximately 0.255 µm (which is equivalent to 83% of the length of a voxel element in our model), the three-phase medium achieves an effective diffusivity comparable to that observed in brain tissue. At this particle radius, the *net* effect of the ECM plus other ECS molecules is to decrease the fraction of space available to the diffusing tracer from 0.2 to 0.140. Importantly, this does not mean that the ECM is a hard shell that occupies 30% of pore space. Instead, it does mean that the hindrance to diffusion caused by both the ECM and other free-floating ECS molecules must have the same effect on the diffusion of small ions that is had by restricting a diffusing particle from 30% of pore space. When the particle radius is 0.255 µm, the average gap width in the model is 1.47 µm. This width is significantly larger than that reported in property P3, and this discrepancy will be explored in the Discussion and Conclusions section. Further, 21.5% of the cells in the three-phase model are concave. This percent should be compared to those models that directly incorporate concavities by punching dead-ends into convex cells [6],[13]. In these models, 100% of the cells must contain concavities to achieve the diffusivity measured in brain tissue.

Further, for one of the proposed models with a lower diffusion coefficient, the effect that must be exerted by the ECM and other ECS molecules would be even smaller. For example, if we consider the model that includes short cuboids and hence has more variation is cell shape and size, we find that the fraction of space available to the diffusing tracer decreases from 0.2 to 0.145, meaning that the hindrance imposed by the ECM and other ECS molecules must have the same effect on the diffusion of small ions that is had by restricting diffusion from 27.5% of pore space. Even the 27.5% proposed here is an upper bound, as will be explored in the Discussion section. Further, it is important to note that the net effects predicted by the model do not allow us to tease out the properties of the ECM, such as concentration and diffusivity, that are responsible for the decrease in the diffusion coefficient. With more biological data, the model can be moved from this first-order approximation to a more realistic representation of the third phase. Even with this first-order approximation, these results strongly suggest that ECS heterogeneity is an important contributor to the low effective diffusivity of brain tissue.

Our simulation results are compared to the following two-point bounds for 3D, three-phase isotropic media:
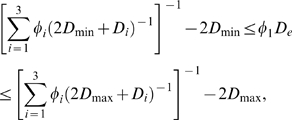
(2)where *D*_max_ and *D*_min_ denote the largest and smallest diffusivities amongst the three phases, respectively [3]. For the example at hand, the three-phase bounds can be greatly simplified. If we let phase 2 be the ICS, then we know that *ϕ*_2_ = 0.8 and *D*_min_ = *D*_2_ = 0 cm^2^ s^−1^. If phase 3 is the ECM, we know that *ϕ*_3_ = 0.2−*ϕ*_1_. Moreover, since we are assuming that the ECM hinders diffusion relative to free diffusion in the ECS, we have that *D*_max_ = *D*_1_ and that *D*_3_ = *aD*_1_, where 0≤*a*≤1. For this situation, the bound in (2) becomes
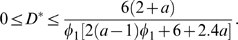
(3)


The upper bound given in Equation 3 is maximized (for any 0≤*ϕ*_1_≤0.2) at *a* = 1, that is, when the ECM (plus other ECS molecules) phase behaves exactly as the ECS and does not act as a hindrance to diffusion. For the special case of *a* = 1, the bound reduces to the two-phase HS upper bound evaluated at *ϕ*_1_ = 0.2: 0≤D*≤0.71, i.e., the diffusion coefficient of the proposed three-phase medium obeys the same upper bound as any isotropic two-phase medium with the same porosity.

## Discussion

The diffusion properties of brain tissue depend on brain cell size, shape and arrangement, as well as dead-end microdomains and ECS heterogeneity, caused in part by ECM macromolecules. The relative contribution of each of these factors is unknown, making it a challenge to develop a realistic 3D model of the brain microstructure. Previous theoretical work in this field resulted in the either: (1) the development of two-phase models that do not achieve the diffusion properties of brain tissue or (2) the development of two-phase models that achieve the observed tortuosity at the expense of violating one or more biological constraint. In this work, we propose a three-phase, biologically constrained (properties P1–P4) representation of the brain microstructure that is consistent with experimentally measured diffusion properties.

In order to justify the development of the first three-phase brain tissue model, it is essential that we elucidate the limitations of biologically constrained two-phase models. To this end, a comprehensive literature search of two-phase models that satisfy the biological constraints was conducted, and we developed a novel two-phase representation of the brain microstructure. Comparing our model to previously proposed models and other personal attempts, we believe that the packing construction used to generate our model gives rise to the most tortuous two-phase model of brain tissue that can be developed, given the constraints specified in properties P1–P4. In this model, both cellular obstacles and ECS channels can vary in shape and size. Despite the variation observed in the model, most of the cells are convex (P1) and ECS channel width does not vary wildly (P3). In both 2D and 3D, this model proved to be more tortuous than models of uniformly spaced convex cells. The importance of dimensionality is highlighted in our two-phase model, as the model was sufficiently tortuous in 2D, but not in 3D. In light of this observation, we conclude that a key mechanism is absent in 3D biologically constrained two-phase models of the brain microstructure.

We propose that conventional brain tissue models must incorporate a third phase, the ECM plus other ECS molecules, in order to be valid representations of the brain microstructure. This proposition naturally follows from biological data that shows that the ECS is a heterogeneous solution with free diffusion properties different than water [20]. The ECS is known to contain a number of glycosaminoglycans, glycoproteins and proteoglycans that constitute the ECM [2]. Although the precise ECM concentration and the effect the ECM has on the diffusion of small ions is currently under debate [11], it has been observed that a charge-interaction effect likely slows down diffusing ions in the ECM and that changes in ECM content occur in brain states characterized by altered diffusion properties [2],[11]. Together, these observations suggest that the ECM impacts the movement of substances in the ECS.

To account for the effects of interstitial composition, others have proposed that the effective diffusivity be written as a product of an exclusion-volume term and an interstitial structure term [7],[23],[26]. Importantly, this is only true if these two effects are independent of one another. However, since the ECM does, to some extent, influence both the interstitial composition of the ECS and the ECS geometry (thus influencing the exclusion-volume term), the assumption of independence does not apply in this case. Furthermore, it has been shown that the effective diffusivity can only be accurately expressed as an infinite series involving integrals over the *n*-point correlation functions [3], and therefore decomposing the diffusivity into a product of geometric and interstitial effects has no rigorous basis. In our model, we avoid this erroneous simplification and treat the ECM (plus other ECS molecules) as the third phase of brain tissue. By directly incorporating the third phase, we can bypass the concern of the theoretically appropriate way to decompose the effective diffusivity. To elaborate, it is not that we avoid considering the infinite series, but that we are measuring the effective diffusivity via a simulation technique that allows us to capture this information without directly measuring the correlation functions. This is comparable to the fact that the effective diffusivity can be accurately ascertained experimentally without considering the precise value of each correlation function.

Simulations validate that the incorporation of the ECM into biologically constrained microstructural models can give rise to an order of magnitude decrease in the diffusion coefficient. Moreover, the diffusion properties of the brain are achieved at the appropriately chosen level of ECS heterogeneity (Figure 3B). The model predicts that that the hindrance imposed by the ECM and other ECS molecules must have the same effect on the diffusion of small ions that is had by restricting diffusion from approximately 27.5% of pore space in order for the appropriate diffusion coefficient to be achieved. Again, this percent is really an upper bound on the effects of the ECM, as the ECM is one of several factors that may be responsible for the low effective diffusivity measured in brain tissue. As previously discussed, structurally complex glial processes have the potential to form dead-space microdomains which increase the brain's tortuosity [11].

Geometrical considerations aside, it is also plausible that current experimental procedures are underestimating the diffusion coefficient in the brain. For example, the finite size of the diffusing ion used in RTI experiments may be a factor responsible for the high tortuosity measured in brain tissue [27]. If the ratio of the radius of the diffusing ion to the average ECS channel width is sufficiently large, the diffusing particle will be prohibited from exploring some of the pore space and the experiment will report a higher tortuosity than is actually found in brain tissue. This exact strategy was exploited by Thorne and Nichoslon [23] to measure the average ECS channel width. A commonly used ion in RTI, TMA^+^, has a radius of approximately 0.56 nm [28]. Taking the average ECS channel width to be 51 nm (the average of the lower and upper bound given in property P3), we can conclude that the diffusing ions are small compared to the size of the pore space, and that finite size effects should not result in a significant overestimation of ECS tortuosity. This reasoning also justifies our use of a point-particle in our first-passage-time simulations. Another experimental factor that must be accounted for is that the RTI tracer can be transiently immobilized upon binding to a surface membrane [2],[13]. Since this action is not accounted for in the calculation of the effective diffusivity, this may cause RTI experiments to report a diffusion coefficient that is lower than what is actually found in brain tissue.

Another point which deserves discussion is the length scales in our model. In the proposed three-phase model that achieves the diffusivity of brain tissue, the average ECS width is 1.47 µm, which overestimates the actual value by two orders of magnitude (property P3). However, this apparent weakness is found in all models of the brain microstructure. Particularly, any model consisting of convex cells [7]–[9], along with models that allow cellular concavities to persist [6],[13] suffer from this same shortcoming. This problem naturally arises because of the large percent of space occupied by the ECS in conjunction with the very small ECS width. Moreover, it is plausible that the width measured in [23] is in some sense an “effective width” in that it incorporates the impact of the ECM and other molecules in the ECS. By no means has this statement been validated, but it is a possible mechanism that may explain some of the discrepancy between the width of the ECS in our model and that measured experimentally, although it certainly would not account for a two orders of magnitude effect.

### Conclusions

We have demonstrated that including the extracellular matrix in a novel brain tissue model composed of nonuniformly spaced mostly convex cells can give the decrease in the diffusion coefficient necessary for the proposed model to conform to the macroscopic properties of brain tissue. This is strong evidence that realistic microstructural models of the brain must account for the effects of the ECM. While the role of dead-ends is minimized in our model, our work does not contradict models that emphasize the importance of dead-ends. Instead, each model probably offers part of the picture, and it is probably some combination of both models that best represents the actual structure of the brain. The contribution of the present work is to give a novel way to consider the effects of the ECM and other ECS components, as well as to propose a novel packing procedure for cells in the brain.

It has been demonstrated rigorously that diffusion properties of a heterogeneous medium can be linked to seemingly different properties of the same medium, including the elastic moduli [29],[30], electrical conductivity [31], and fluid permeability [32],[33]. In a future work, we will examine such *cross-property* relations [3] for model brain microstructures.

## Methods

We use a modified Brownian motion simulation to determine the effective diffusivity of random media [21],[34]. In a standard Brownian motion simulation, the detailed zig-zag motion of a point-sized random walker is modeled for a finite number of steps, where the step-size of the walker is (in theory, infinitesimally) small. The dimensionless effective diffusivity D* of the medium can be obtained by averaging the long-time behavior of the mean-square displacement of many diffusing Brownian particles.

**Figure 4:**
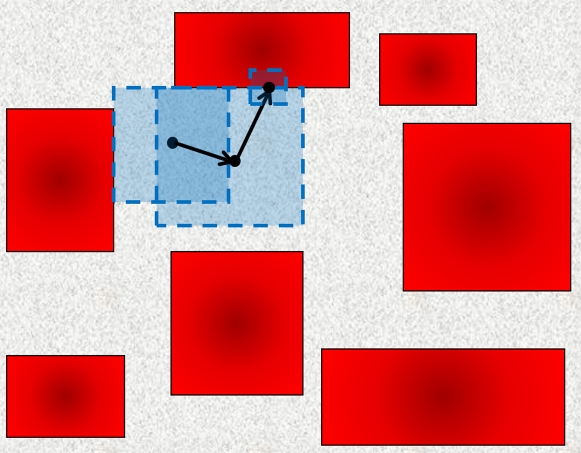
First-Passage-Time Algorithm. Example of random walk in 2D using first-passage squares.

This random walk approach can be considerably sped up by using first-passage-time equations [21]. To implement this technique, at each step of the simulation a bounded region surrounds the random walker. The walker jumps onto the surface of this first-passage region in one step (Figure 4), provided that the probability to first hit the surface at a given location and the associated average hitting time are known. A single jump onto the first-passage surface is equivalent to taking many small steps in a standard random walk algorithm, and hence the first-passage-time technique executes significantly faster than standard random walk algorithms [21]. The efficiency of the algorithm is highly dependent on the volume fraction of the higher diffusivity phase (i.e., pore space) and the level of discretization of the region. However, one of the advantages of the algorithm is that at a fixed volume fraction and discretization level, the efficiency of the algorithm is not significantly impacted by the geometric complexity of the material being studied, hence making it especially useful for studying complex materials.

Both continuous and discrete first-passage-time techniques have been developed and applied to determine the effective conductivity of equilibrium distributions of hard disks [35], hard spheres [36], overlapping spheres [37], and hard ellipsoids [38], as well as to determine effective properties of digitized images [39]. Since we have developed digitized representations of brain tissue, only the discrete first-passage-time algorithm is described herein, and we choose to only explain the algorithm with a point-sized diffusing particle. The generalization to a finite-sized particle is straightforward, with the idea being that the obstacles are made larger to compensate for the finite size of the diffusing tracer. Details of this algorithm can be found in [25].

### Discrete First-Passage-Time Technique

Consider a Brownian particle diffusing in a two-phase digitized composite material consisting of pixels (2D) or voxels (3D) which either have finite diffusivity *D*_1_>0 (representing the ECS) or *D*_2_ = 0 (representing the ICS). In order to cope with the computational limitations of modeling a very large region of brain tissue, the algorithm employs periodic boundary conditions. For digitized images, the natural first-passage region is determined by the shape of a pixel and voxel; that is, squares are used in 2D and cubes are used in 3D [39]. In order to simulate the diffusion of a particle in a 3D digitized composite, the following set of rules [39] is applied:

1. Introduce the Brownian particle into a random phase 1 voxel (with *D*_1_>0).
2. While the walker is sufficiently far from the two-phase interface, construct the largest possible homogeneous cube centered at the Brownian particle. Define half the length of the cube to be *L*.
3. In one step, the random walker jumps to a point on the surface of the cube (Figure 4). First, the walker randomly chooses to move to one face on the cube. The location the walker moves to on this face, (*q*,*p*), is chosen from the following probability distribution [39]

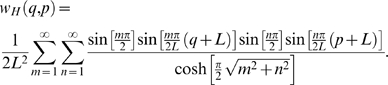
(4)Torquato et al. (1999) have shown that the time associated with moving to a point on this homogeneous first-passage cube is given by
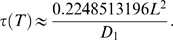
(5)
4. If the walker is within some prescribed very small distance of the two-phase interface, construct a first-passage cube that overlaps the interface (Figure 4). Let each octant of the heterogeneous first-passage cube have the constant diffusivity *D* ^(*i*)^, and let (*q*,*p*) represent the boundary coordinate on any face of the first-passage cube. The location of (*q*,*p*) is chosen from the following probability distribution [39]

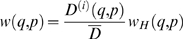
(6)where 
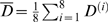
 is the average diffusivity of the first-passage cube. It is important to note that this distribution is actually a piecewise function, as *D* ^(*i*)^ can take on different values in each quadrant of the first-passage cube's face. Given this distribution, the probability of moving to any face *ν* of the heterogeneous first-passage cube is given by [39]

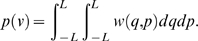
(7)The time taken to move to a point on the heterogeneous first-passage cube is 
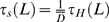
, where *τ_H_*(*L*) denotes the homogeneous solution given in Equation 5 for unit diffusivity [39].
5. The algorithm is run until we can be (c*100)% confident that the actual value of D* falls in the range [D*−*δ*, D*+*δ*]. This is implemented by repeating the simulation for *N* random walkers until:
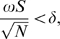
where *S* is the sample standard deviation and *ω* solves:
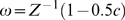
with *Z* being the standard normal distribution [40]. We chose to use *c* = 0.95 and *δ* = 0.001 in our simulations in order to ensure convergence.

The details of the analogous 2D algorithm can be found elsewhere [39].

### Calculating the Effective Diffusivity

In any dimension *d*, the dimensionless effective diffusion tensor of the medium, 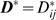 is given by [38]

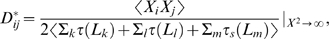
(8)where the values of *X_w_* (displacement in the *w* ^th^ direction) and *τ* (time taken to hit the surface of a bounding region) are calculated using the first-passage-time algorithm described above. The summation over the subscript *k* denotes Brownian paths in phase 1, over *l* denotes paths in phase 2, and over *m* denotes paths at the two-phase interface.
