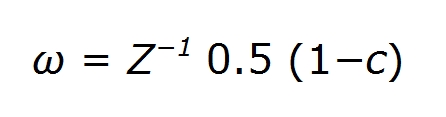# Correction: A Novel Three-Phase Model of Brain Tissue Microstructure

**DOI:** 10.1371/annotation/c9faa83b-3c7b-4f38-8d74-1a4309403688

**Published:** 2009-01-06

**Authors:** Jana L. Gevertz, Salvatore Torquato

In the Methods section, there was an error in the second equation in the fifth rule of the section titled "Discrete First-Passage-Time Technique." The quantity in parentheses is incorrect. It should read 0.5(1-c). For the complete, correct equation, see here: